# Asymmetric Neonatal Spasms As an Early Sign of Brain Malformation Potentially Caused By Regular Light Alcohol Consumption During the First 22 Weeks of Pregnancy, a Clinical Case Report

**DOI:** 10.34763/devperiodmed.20192301.1520

**Published:** 2019-04-08

**Authors:** Olha Tychkivska, Cristina Go, Yuriy Korzhynskyy, Oksana Ostalska

**Affiliations:** 1 Department of Pediatrics and Neonatology, Lviv National Medical University Lviv, Ukraine; 2 Division of Paediatric Neurology, Epilepsy and Clinical Neurophysiology, the Hospital for Sick Children, University of Toronto, Toronto, ON, Canada; 3 Department of Pediatrics and Neonatology, Lviv National Medical University Lviv, Ukraine; 4 NICU of Lviv City Children’s Hospital Lviv, Ukraine

**Keywords:** epileptic spasms, seizures, cerebral malformation, neonatal period, light alcohol drinking

## Abstract

**Introduction:**

Epileptic spasms are seizures usually associated with a severe developmental epilepsy syndrome with onset in the first year of life, peaking between 3 and 1O months of age [12]. A variety of disorders can cause epileptic spasms, with the etiology driving management, prognosis, and overall outcome. Preexisting brain damage has been demonstrated in 60% to 90% of the cases reflecting pre-, peri-, or postnatal brain injury that may usually be determined by history and clinical neurologie examination. Cerebra/ malformations may account for up to 30% of the cases [2]. Prenatal a/coho/ exposure can permanently damage the brain, affecting important structures, such as the cerebellum, corpus callosum as we/I as specific cell populations in many other regions of the brain. No one knows what a "safe" amount of a/coho/ consumption du ring pregnancy may be [3].

**Objective:**

The aim of this article is to present a clinical case of a large brain tempora/ lobe malformation which was recognized after a very early onset of spasms registered on video EEG-monitoring followed by MRI findings and to put forward the assumption that regular consumption of light alcoholic drinks even in low doses could con tribute to irreversible brain damage in the fetus.

**Material and methods:**

All patient data were collected from the N/CU and Newborn Patho/ogy Department of Lviv City Children's Clinical Hospital Health Record Department, and included the hospital and clinic records by the staff neurologist, neurophysiologist, and pediatrician, as we/I as EEG records in the postneonatal period. The mother was interviewed to clarify the pregnancy course data. The mother's consent was obtained for publication.

**Results:**

Asymmetric spasms, which were recognized as seizures on the 4^th^ day of the child's life white recording video EEG, urged the physicians towards further diagnostic investigations. Primarily the child was diagnosed with neonatal abstinence syndrome on the 2^nd^ day of life based on clinical and patient history data, but on the following day episodes of myoclonic jerks and jitteriness were noticed and video EEG monitoring started. Upon analysis of video- EEG, myoclonic seizures and spasms were reported showing asymmetry in the amplitude of ictal EEG. MRI was recommended and performed to explain focal EEG findings, and a large brain left temporal lobe malformation was seen.

**Conclusions:**

Spasms in the form of seizures are rarely reported in the neonatal period. Their recognition has to lead to urgent brain imaging study to look for the underlying cause and to implement timely, appropriate corrections in the treatment strategy. Although brain malformations can have many causes, taking careful antenata/, perinatal and family history has ruled out many usual etiologies. Materna/ alcohol consumption during pregnancy may potentially have contributed to the condition.

## Introduction

Epileptic spasms (ES) are sudden and brief contractions of axial (neck and trunk) and proximal limb muscles. ES may involve flexor or extensor muscles, and both patterns can be present in the same child depending on whether the child is sitting or is in a supine position. In contrast to other types of motor seizures that are derived from specialized cerebral cortical motor areas, signs such as head, or eye deviation, one-side facial, limb, hand or foot contractions, are rarely observed during the ES [4]. An ES can be an isolated event or, more typically, occurs in a cluster [2], mainly on awakening or during transition from slow NREM sleep to REM sleep. Sometimes ES may be subtle and consist of upward eye deviation or slight shoulder elevation only. Most commonly, spasms show symmetric behavioral and EEG manifestation, but in 10% to 30% of the patients with spasms they are asymmetric. The length of muscle contractions may vary: most ES are characterized by a phasic contraction lasting 1 to 2 seconds (spasm seizure), others may have the same initial phasic component, followed by less intense but more sustained tonic contractions of variable duration, lasting up to 10s (spasm-tonic seizure) [4]. In the 2017 ILAE Seizure Classification, epileptic spasms were confirmed as a type of seizure, with onset defined as unknown, generalized or partial [5]. ES is the type of seizure which characterizes infantile spasm (IS) syndrome and West syndrome [4]. The incidence of IS syndrome ranges between 2.9 and 4.3 per 10,000 live births. The age of onset of IS peaks between 3 and 9 months. An earlier beginning and a later onset have been reported, usually associated with a symptomatic etiology [4]. A new concept in IS etiology classification has been proposed: instead of the terms: cryptogenic, idiopathic, symptomatic, it was recommended to classify them as 1) genetic, 2) structural-metabolic, and 3) unknown cause6.

Prenatal causes include: congenital malformations, congenital infections, neurocutaneous disorders, chromosomal, metabolic disorders, and congenital syndromes. Perinatal causes include hypoxic-ischemic encephalopathy, and hypoglycemia. Some postnatal causes are: meningoencephalitis, stroke, trauma, hypoxic-ischemic stroke, tumors [2]. Ictal EEG of IS usually shows high amplitude slow waves, which are usually diffuse. A brief beta activity (spindle-like) or very fast rhythmic activity may proceed or/ and superimpose on the slow waves, at least in their ascending part. Interictal EEG IS pattern usually indicates hypsarrhythmia, but there were cases reported when infants never developed this pattern.

Cerebral malformations are a major cause of IS which usually start early, in the first year of life. Among the cerebral malformations, disorders of abnormal neurogenesis, neuronal migration and neuronal organization have been reported [4]. Focal cortical dysplasia (FCD) is one major cause of IS (symmetric or asymmetric), but is very hard to recognize on MRI in neonatal and infancy periods due to poor myelination of the cerebrum at that age [12].

Recognizing malformations of cortical development based on neuroimaging results (MRI, PET, SPECT) improves treatment options, and helps in the prognostication of patients with IS, or West syndrome4.

Baseline EER, MRI, PET and neurological examination of children with asymmetric spasms reveal structural or functional abnormalities in the contralateral central region significantly more often in the children with >50% spasm asymmetry or asynchrony than in other children. Asymmetric spasms, hemihypsarrhythmia and partial seizures combined with IS have a significant association with asymmetric brain pathology and findings also suggest that the generator of asymmetric and asynchronous spasms involves the primary sensorimotor cortex [10, 11].

The choice of 1st line therapy for IS can be guided by the etiology: vigabatrin is more effective in cortical dysplasia, tuberous sclerosis, whereas hormonal therapy is more effective in HIE and cryptogenic forms. Similarly, the long-term cognitive outcome of IS is mostly etiology-dependent [4].

Excessive alcohol exposure during pregnancy can cause damage to the child during all stages of prenatal development (preimplantation, embryonic, fetal) [2, 7]. There are many individuals who fall into the spectrum of FASD who do not exhibit the classical phenotypic features of fetal alcohol syndrome but whose prenatal alcohol exposure has adversely affected brain development, with an estimated prevalence in the general population of approximately 1%. These alcohol-associated brain abnormalities include aplasia or hypoplasia of the corpus callosum, cerebellar abnormalities with interglobal hypoplasia or vermian dysgenesis, reduced cortical thickness [8], heterotopias, widespread cortical and white matter dysplasias, and defects of neuronal and glial migration9.

## The aim

The aim of this report is two-fold: to emphasize that identifying seizure semiology through clinical observation and continuous video-EEG (cEEG) monitoring might be a key point in the diagnostic process of the underlying condition. In this article we demonstrate this through a case report of a newborn with epileptic spasms, the type of seizures that are very rare in the neonatal period, identified during the first few days of life. This prompted an urgent brain MRI for diagnosis of the possible etiology, even though head ultrasound and the CSF test were normal. The secondary aim of this report was to emphasize that alcohol consumption can potentially cause permanent damage to a baby’s brain and that no one knows what a “safe” amount of alcohol consumption during pregnancy may be.

## Materials and methods

The patient’s medical history data were collected from the NICU and Newborn Pathology Department of Lviv City Children’s Clinical Hospital Health Record Department, and included the hospital admission and subsequent clinic follow-up notes by the staff neurologist, neurophysiologist, and pediatrician, the interview notes with the mother, as well as EEG records in the postneonatal period. EEG-monitoring data was recorded by 4-channel video-EEG DigiTrack CFM device in referential montage (F3, F4, P3, P4, with Czas reference).

## Results

The child was admitted to the NICU department of Lviv City Children’s Hospital on the 2nd day of life with jaundice, generalized weakness, sluggish reaction to stimuli, and jitteriness.

From the patient’s medical records, it was known that the boy was born preterm to a 17-year old single mother (Gravida 1, Para 0), by fast, spontaneous vaginal delivery at 36 weeks of gestational age, with birth weight of 1950g, and height of 46cm. The fluid was meconium-stained, and the APGAR scores were 8 and 8. From the mother’s and maternal grandmother s account, the mother did not know she was pregnant until the 18th week of pregnancy. Before and during the first 4 months of pregnancy she worked in an alcoholic beverages factory, responsible for labeling bottled drinks. She used to have one or two (250-500 ml) low dose (Alcohol Percentage Content: 2.5-6%) alcoholic beverages per day, 3-6 days per week till the 22nd week of her pregnancy. The pregnancy course was complicated with threatened abortion in the 27th week

The mother’s blood group was O (I) Rh+, and the newborn’s - A(II)Rh+. The level of indirect bilirubin in umbilical blood was 48.8 pmol/1, haemoglobin – 156 g/1, RBC- 4.68x1012/l. Besides episodes of recurrent tremor during the first 18 hours of life, the child’s condition was stable. Towards the end of the first day of life, jaundice appeared and quickly spread from the facial skin and sclera to the body and skin on the limbs. The level of indirect bilirubin increased to 237 pmol/1, the level of direct bilirubin was 10.9 pmol/1 with haemoglobin of 150 g/1, RBC – 4.5x1012/l. Hemolytic disease of the newborn (icteric type, *ABO incompatibility*) was diagnosed and phototherapy started. Nevertheless, the child’s condition continued to deteriorate because of generalized hypotonia, including sucking weakness, hyporeflexia, and more frequent episodes of jitteriness followed by the child’s prolonged crying episodes that were hard to manage. The decision was made to transfer the child to NICU Department of Lviv City Children’s City Hospital with suspected neonatal abstinence syndrome and mild to moderate hypoxic-ischemic encephalopathy.

On admission to NICU, the child was awake, had a moderate generalized muscle weakness, hyporeflexia, recurrent episodes of tremor accompanied by prolonged periods of crying and irritability, the skin of the face, trunk, limbs besides the palms and soles was yellow. The level of indirect bilirubin was 250 pmol/1, the level of direct bilirubin was 10.0 pmol/1 with haemoglobin of 150 g/1, RBC- 4.38x1012/1, leukocytosis 25,5x109/1, neutrophils – 65%. Besides haemolytic disease of the newborn (icteric type, *ABO incompatibility)*, perinatal infection, HIE and neonatal abstinence syndrome could still not be ruled out. Nevertheless, urinalysis was normal, biochemical inflammation markers, RW, HIV, HBV, Hbs Ag, and TORCH PCR in the blood came back negative. CSF analysis revealed no pathological changes. Electrolytes, glucose, transaminase, creatinine, urea blood levels were also normal. The direct Coombs test came back negative, reticulocytes – 0.9%. Urine and blood cultures were negative as well. Ultrasonography of the brain and internal organs did not reveal any pathological findings. Echocardiography revealed PFO 2 mm. All ECG variables were within normal ranges for the age of the child. The mother denied taking illicit drugs during pregnancy and her urine drug tests came back negative.

**Fig. 1:**
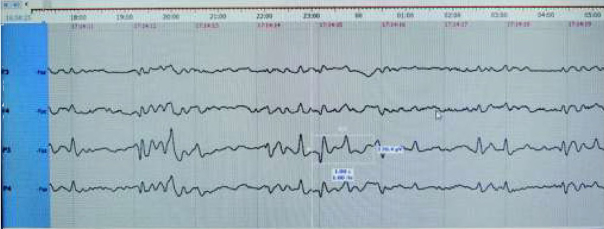
lctal EEG showing repetitive sharp and slow waves (sharp wave corresponds to myoclonic jerk), predominantly in P3 and P4 (with higher amplitude in P3). Rye. 1. Ictal EEG pokazuje powtarzające się ostre i wolne fale (fala ostra odpowiada mioklonicznemu szarpnięciu), głównie w P3 i P4 (z wyższą amplitudą w P3).

**Fig. 2:**
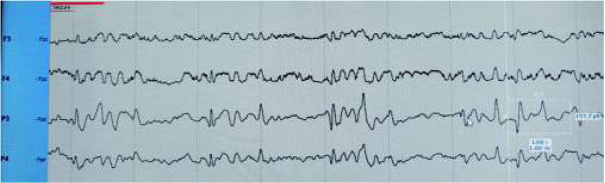
Ictal EEG showing repetitive sharp and slow waves (sharp wave corresponds to myoclonic jerk). Rye. 2. lctal EEG pokazujący powtarzalne ostre i wolne fale (ostra fala odpowiada szarpnięciu mioklonicznym).

Intensive phototherapy was continued, and bilirubin levels slowly went down, but at the beginning of the 3rd day, the child was noticed to have myoclonic jerks of the lower limbs, predominantly starting with jerking of the right leg and followed by the left leg. Myoclonic jerks periodically occurred in clusters. The decision was made to start cEEG monitoring. Ictal EEG showed arrhythmic repetitive sharp waves associated clinically with myoclonic jerks and post jerk slow waves (fig. 1, 2).

EEG between the episodes of jerks was normal.

**Fig. 3:**
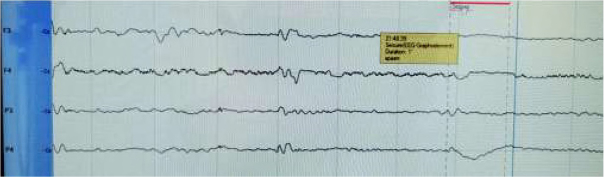
Spasm (marked as seizures in the picture) accompanied with a slow wave and postictal suppression of the background activity. Ryc. 3. Spazm (oznaczony jako drgawki na obrazie), któremu towarzyszy powolna fala i poudarowe/ponapadowe tłumienie aktywności tła.

**Fig. 4:**
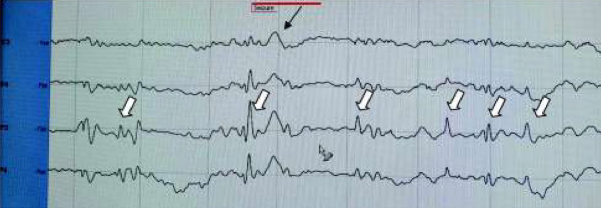
Myoclonic seizures (marked with 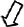), and spasm (marked with ↙ and sign "seizures"). Ryc. 4. Napady miokloniczne (oznaczone 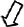) i skurcz (oznaczone ↙ napisem "seizures").

Besides myoclonic jerks, and prolonged episodes of jitteriness, cleg also helped to reveal one more type of seizure – spasms: sudden, brief flexor contractions of the neck and proximal parts of limbs that electrographically was accompanied by a slow wave, periodically with the following suppression of the background activity (fig. 3, 4).The child was loaded with Phenobarbital (PB) 20mg/ kg (oral, as parenteral form is not available in Ukraine) and then kept on a maintenance dose of 7 mg/kg, which helped to stop clinical myoclonic seizures and jitteriness completely during 18 hours after the therapy was started.

Keeping in mind that the spasms might need a specific treatment, the child was kept on cEEG for 5 more days after PB was initiated, but after the 2nd day of PB no more spasms were registered on EEG. The plan to start hormonal therapy with prednisolone and vitamin B6 was deferred. An urgent MRI of the brain was performed due to the left-sided predominance of ictal findings on the EEG.

**Fig. 5:**
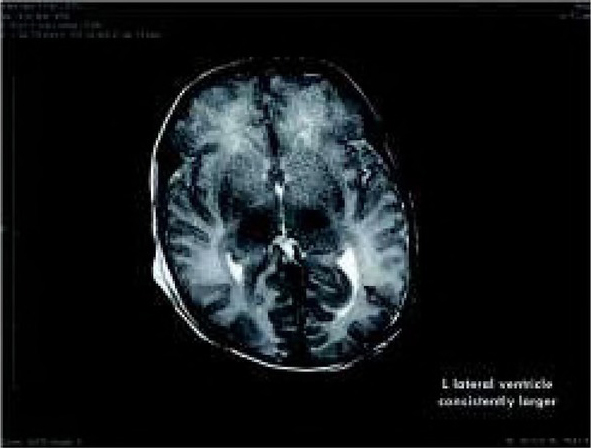
Brain MRI of the patient, arrow points to the affected left side. Ryc. 5. MRI mózgu pacjenta, strzałka wskazuje na uszkodzoną lewą stronę.

Brain MRI performed at day 7 of life revealed that the left temporal horn of the lateral ventricle was consistently larger than the right side (there might have been an early injury of the brain in that area. Another possibility is a brain malformation in that area that is not very visible yet because of the age), no signs of HIE or inflammatory process were seen (fig. 5).

Concordant cEEG-video monitoring and brain MRI findings helped to make the correct etiologic diagnosis of the large left temporal lobe structural abnormality which corresponded with clinical seizures. The case was discussed via telemedicine with colleagues at the Hospital for Sick Children (Toronto, CA), with cEEG and MRI data reviewed, and the diagnosis was confirmed.

## Discussion

The high index of suspicion for clinical seizures and further investigations using cEEG-video monitoring and brain MRI together with careful prenatal, perinatal and postnatal history-taking helped to determine the definite diagnosis of left temporal lobe brain malformation and to explain the neurological-state deterioration at the time when no signs of infectious process, moderate or severe HIE were revealed and the indirect bilirubin level was decreasing, while the child was being administered intensive phototherapy. As no other antenatal and prenatal factors were noted in the medical records, we may assume that the prolonged period of light alcohol beverage consumption before and during the first four months of pregnancy affected the developing brain. The child’s mother confirmed that she continued drinking light beer in small amounts (100-150-200 ml per day) 2-3 days per week as she did not consider this amount to be dangerous for her baby. Though epileptic spasms are very rare at this age, the presence of brain malformation probably produced that type of seizure, which occurred with a specific ictal pattern seen during the epileptic spasms, even though interictal hypsarrhythmia was not seen. Even a limited 4-channel cEEG module plus CFM (aEEG) helped to reveal seizures and to recognize the seizure types correctly. Oral prednisolone and vitamin B6 therapy were discussed as potential treatments for the spasms, but were never started because Phenobarbital helped to achieve a full control of the seizures, confirmed by further cEEG monitoring for several days after treatment was started. Currently, the child has been seizure-free for 6.5 months, taking 4.5 mg/kg/d of Phenobarbital. The follow-up routine EEGs, recorded once a month after discharge, revealed intermittent sharp waves in the left temporal and parietal areas, periodically showing a field at the right parietal and temporal regions. The child's physical and psychomotor development corresponds to the age. Because of the presence of brain malformation, the child is seen by a neurologist and pediatrician monthly, and EEG recording is made every 1.5-2 months to make sure there will be no transition of interictal EEG into hypsarrhythmia and to monitor clinically for spasm recurrence.

## Conclusions

Spasms are not typical seizures in the neonatal period, therefore the appearance at this age needs a further precise investigation of the possible underlying condition. A thorough clinical history, physical examination, EEG monitoring and careful review of neonatal brain MRI are very important tools to help arrive at the correct diagnosis which significantly impacts the proper choice of seizure management and the underlying condition. Alcohol consumption during pregnancy can cause irreversible changes in the developing fetal brain and other organ systems. This is why women who are contemplating pregnancy or are pregnant should abstain from drinking alcohol.
